# Embedding consumer and community involvement within an established research centre: moving from general recommendations to an actionable framework

**DOI:** 10.1186/s40900-020-00241-2

**Published:** 2020-10-27

**Authors:** Tilini Gunatillake, Cade Shadbolt, Daniel Gould, Michelle Lam, Marion Glanville Hearst, Carol Vleeskens, Peter Choong, Michelle Dowsey

**Affiliations:** Department of Surgery, The University of Melbourne, St Vincent’s Hospital Melbourne, Melbourne, Australia

**Keywords:** Consumer, Community, Consumer engagement, Consumer involvement, Orthopedic, Musculoskeletal, Arthritis, Research

## Abstract

**Plain English summary:**

Involving consumers and community members in the research process is an important step towards developing and delivering effective, person-centered health care. The National Health and Medical Research Council have provided recommendations for involving consumers and community members in research; however, definitive actions to implement these are not well defined.

To address this, an established research centre in Melbourne, Australia, has developed a consumer and community involvement framework to incorporate the national recommendations into their research program. This paper describes the framework the research centre has employed, in the hope that other researchers can adapt this approach and learnings to their own research practices.

The framework described in this paper aims to foster partnerships between consumers, community members and researchers, and in doing so, encourages consumers to be actively involved in research to help improve future outcomes for those living with musculoskeletal conditions. Simultaneously, the framework encourages researchers to value the consumer voice in their research to ensure they yield meaningful research outcomes for those living with musculoskeletal conditions.

**Abstract:**

**Background**

The value of involving consumers and community members in every stage of the research process is gaining recognition as an important consideration in the wider research landscape. The National Health and Medical Research Council (NHMRC) has provided general recommendations for involving consumers and community members in research, although the translation of these recommendations into tangible actions has not yet been well defined. In light of these recommendations, many research institutions are now seeking to incorporate the voices of consumers and community members in their research practices.

**Methods**

The consumer and community involvement framework described in this paper incorporates the NHMRC’s recommendations to produce a four-tiered model where consumer participants nominate their level of involvement depending on their research interests and preferred level of commitment. In ascending order, the tiers are: Consumer Subscriber, Document Reviewer, Research Buddy and Consumer Advocate.

The success of this framework depends upon the implementation of effective governance and access to appropriate infrastructure. A Consumer and Community Advisory Group and a designated Consumer and Community Liaison Officer will take responsibility for ensuring appropriate interactions between consumers, researchers, and the research center’s executive team. The framework aims to apply suitable support structures in place to manage expectations and minimize barriers to effective involvement, whilst ensuring that consumer contributions are appropriately valued and incorporated in the research.

**Discussion**

Involving consumers and community members in the research process is an important step towards developing and delivering effective, person-centered health care. While consumer and community involvement offer researchers invaluable perspectives on their research program, it provides an opportunity for consumers and community members to be actively involved in health research and improve the health and wellbeing for those living with health conditions.

## Background

The voices of consumers and community members are increasingly shaping the research landscape. This shift is in response to the call to action by academic journals, as well as research, policy, and funding bodies to engage community members and healthcare consumers in all areas of medical research. Well-implemented consumer and community involvement (CCI) initiatives benefit the wider public by ensuring research outcomes are relevant to the needs of the community, which is critical to the translation of research into practice [1]. CCI is also crucial for securing funding, disseminating findings, and increasing research impact [1, 2].

In partnership with the Consumer Health Forum of Australia, the NHMRC has outlined its vision for CCI in the *Statement on Consumer and Community Involvement in Health and Medical Research* [1] (herein referred to as ‘the Statement’). In addition to re-emphasizing the NHMRC’s commitment to CCI, the Statement also provides general information about practical ways the Statement can be implemented by research institutions, researchers, consumers, and community members. The Statement focuses on actionable, pragmatic steps in the process of developing and implementing CCI programs. However, it cannot account for the nuances and highly contextualized nature of each individual research group. It is, therefore, critical to translate these general recommendations into a context-specific framework that meets the needs of consumers and community with which research institutions are engaged.

There appears to be no single universal model of CCI that can be adapted to suit all research settings [3]. A meta-narrative systematic review determined that there is no comparative data in published patient engagement models within research to propose best practices [3]. Furthermore, a recent analysis of existing frameworks that support CCI in health research identified that the development and success of a program is highly context-specific with some common threads [4]. These included a diverse range of voices within the program, good governance structures, leadership support, training opportunities and evaluation for continuous quality improvement [4]. The issue of tokenistic and tick-box approaches was recognized as a common danger across many consumer involvement programs, whereby research groups merely seek to appear as though they are engaging with consumers without genuine partnership in their research study [3, 4].

In the Australian context, the South Australian Medical Research Institute (SAHMRI) has developed an evidence-based, co-designed framework for consumer and community engagement at their research institute [5]. Their framework details four main domains as integral to the success of consumer and community engagement: governance, infrastructure, capacity, and advocacy. This involves: organization-wide policies recognizing consumers as stakeholders in research; ensuring consumers have access to support networks; and building the capacity of both consumers and researchers to undertake meaningful engagement. Underpinning these domains is the importance of fostering the relationships between consumers, researchers, and the organization [5].

A report by Saunders et al. [2], outlines how the model framework included in a previous version of the Statement was implemented by a philanthropic research funding body. The experiences reported by Saunders et al. provide useful insights into how the latest iteration of the Statement might be implemented in other organizational contexts. However, it is important to note that these insights are ultimately drawn from experiences in a single area of the medical research landscape (i.e. philanthropic research funding) and in this rapidly developing field there has been further development on the implementation of CCI in research as outlined by the NHMRC and SAHMRI [5, 6].

To demonstrate the application of the NHMRC’s recommendations, this paper outlines the approach taken to implement the current Statement into an established research organization. The literature outlines the benefit of creating ones’ own consumer involvement framework, as the success of a program is highly context specific. However, utilizing a range of resources to inform a framework is considered beneficial, as it facilitates learning and provides an example which can be adapted to the local context [4].

By detailing how we have moved from general recommendations to a framework relevant to our needs, we will highlight how we worked to overcome some widely recognized barriers to maintain high-quality consumer involvement. Providing a clear and practical framework with actionable procedures will allow similar research organizations to adapt and devise their own programs.

### Consumer contributions

In keeping with our philosophy and established protocol of embedding consumer and community involvement in all aspects of the research cycle, it is acknowledged that this protocol was developed with valuable contributions from Marion Glanville Hearst and Carol Vleeskens.

Marion Glanville Hearst was a participant in a Mindfulness meditation trial led by our research organization. This trial involved patients awaiting hip or knee joint replacement surgery who were randomized to either standard care or an 8-week mindfulness course prior to undergoing joint replacement surgery. Marion has first-hand experience in our research program and brings along a wealth of experience from her degrees in psychology and medical anthropology. Marion has over 25 years of experience working as an occupational therapist in various psychiatric settings, including, inpatient, community centers, and residential rehabilitation.

Carol Vleeskens is the convener of the Musculoskeletal Clinical Academic Group Consumer Community Council under the auspice of ‘Maridulu Budyari Gumal’, the Sydney Partnership for Health, Education, Research & Enterprise (SPHERE) and has lived experience of musculoskeletal conditions. As a sociologist with post-graduate qualifications in community participation, she has 30 years of experience in the government and not-for-profit sectors within South Western Sydney. Throughout her career she has worked in community development, child protection, addiction services, suicide prevention, health services planning and health promotion, to drive change relevant for their consumer population. In her voluntary role as a consumer representative she has been an active member of the South West Sydney Local Health District’s Community Participation Program since its inception in 2002 as well as acting as an advisor to other Clinical Academic Groups and consumer groups within SPHERE.

### Design

The NHMRC Centre of Research Excellence for Total Joint Replacement, or OPUS (OPtimization of oUtcomes, improved equity, cost effectiveness and patient Selection), recognizes that within the area of orthopedic research in Victoria, there are no formal programs for researchers to incorporate consumer experiences into the inception and design of research projects. We have addressed this by devising a program that incorporates the consumer perspective at every stage possible, beginning at research project design through to research implementation and result dissemination. The OPUS Consumer and Community Involvement Program (from herein referred to as the CCIP) aims to encourage consumers, in partnership with researchers, to improve the quality and integrity of the research conducted.

### The OPUS consumer and community involvement program

**Fig. 1:**
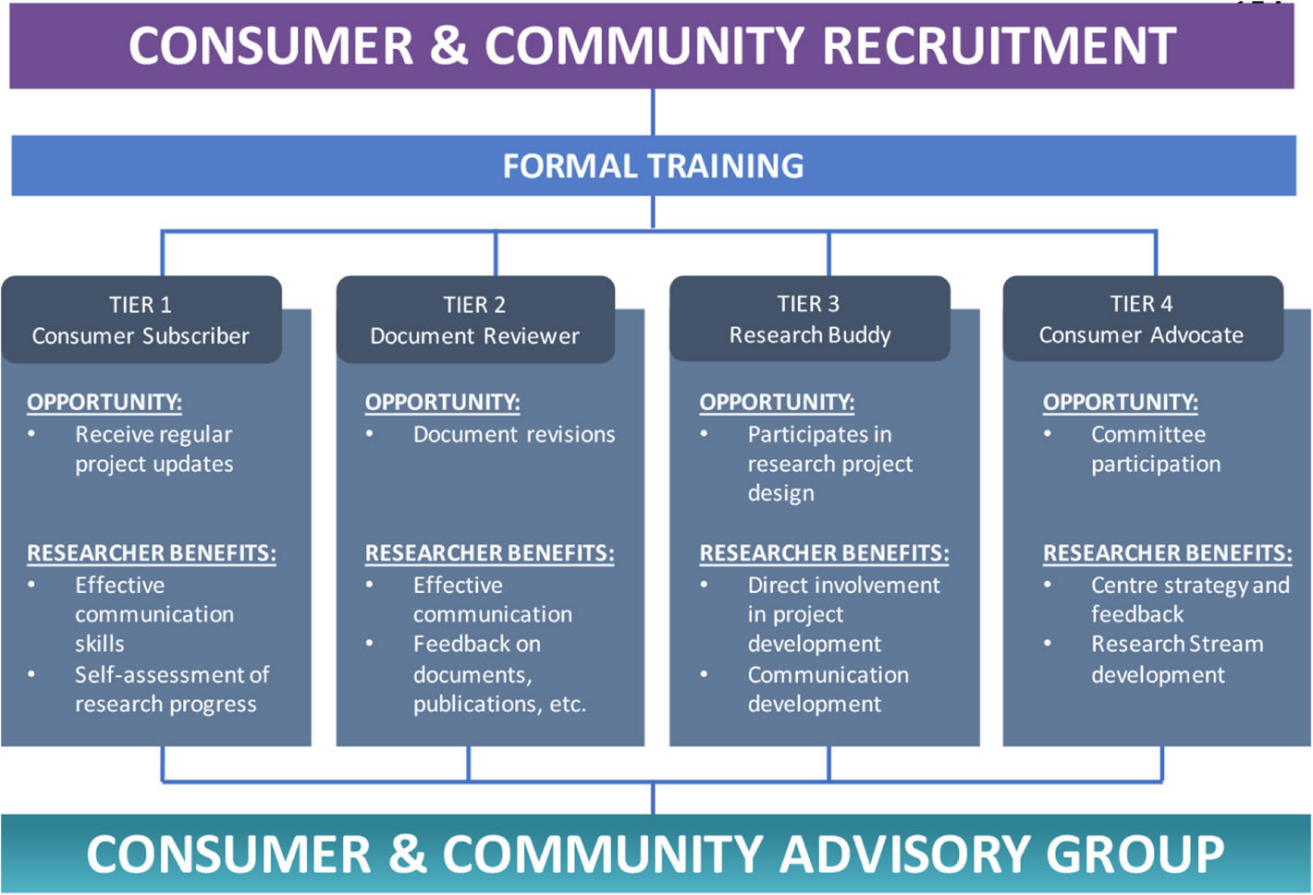
Proposed level of involvement for consumers and community members presents different opportunities of participation and the relevant benefits to researchers

The CCIP uses a four-tiered approach to cater for different consumer requirements (Fig. 1). These requirements are dictated by the participants’ time commitments and desired level of involvement (Table 1).

**Table 1:** Levels of involvement for consumer and community members, expectations and the training required for each Tier

	Level of involvement	Expectation
**Tier 1: Consumer subscriber**	• Receive electronic communications and research progress updates • Minimal research input	• Respond to basic surveys, questionnaires, stay engaged via research updates and newsletters
**Tier 2:** **Document reviewers**	• Review videos, websites, information sheets, surveys, grants, publications, and media releases	• Review and provide feedback for documentation that can vary in complexity
**Tier 3:** **Research buddy**	• Participate in research project design, implementation, evaluation, and dissemination of results • Consumers and community members can engage with researchers and students to understand more about the research conducted, the challenges and the people behind it	• Attend meetings with researchers • Commit personal time to read and prepare for meetings • Provide input and advice on the development of research projects, directions, and grants
**Tier 4:** **Consumer advocate**	• Provide advice as an advocate of the broader consumer/community perspective • Sit on committee meetings and have good understanding of the research projects and broader research themes to provide advice	• Participate in quarterly committee meetings as Chairs • Advise on the consumer experience • Recruit new consumer and community members and mentor new members as appropriate • Develop and conduct training for prospective consumer and community members and for researchers on all aspects of CCI in research

Truly effective consumer and community involvement in research is often undermined by researchers only consulting with consumers in the later stages of research [7]. To ensure OPUS integrates effective involvement as per our ethos, consumers and community members are afforded the opportunity to provide strategic direction over the type of research carried out within the research centre. Participants have the option to adjust their involvement with research (i.e. moving from Tier 2 to Tier 3 level) at any time if they wish to become more involved in projects. Should this occur, the appropriate training and support for the new tier is provided.

### Governance

The *Consumer and Community Advisory Group (CCAG)* oversees the operations of the program. The CCAG meet monthly to discuss the strategic direction of the program, its progress, activities, resolves any issues or grievances and provides safe and impartial support for consumers and researchers. Advisory group membership includes consumer advocates (Tier 4 participants), researchers, OPUS management and support staff (Table 2).

**Table 2:** CCAG membership roles and responsibilities

Role	Member	Experience	Responsibility
Chair	Consumer Advocate	Non-researcher	The Chair must have a good understanding of the program and CCAG purpose, control, and guide meetings effectively. Where appropriate, the Chair will receive formal training to be an effective Chair.
Co-Chair	Consumer Advocate	Non-researcher	The Co-Chair will support the Chair and provide secondary leadership.
Advisor	Consumer Advocate	Non-researcher	An additional Consumer Advocate will sit on the CCAG to balance the strategic direction of the CCIP.
Advisor	Researcher	Researcher	One OPUS researcher will be nominated to the CCAG to provide the researcher perspective.
Advisor	PhD Student	Researcher	One PhD student will be nominated to the CCAG to represent student cohort perspective.
Advisor	OPUS Executive	Researcher	A member of the OPUS Executive Committee will sit within the CCAG to advise on major initiatives and strategic direction.
Administrative support	Consumer and Community Liaison Officer (CCLO)	Non-researcher	Secretariat support, communications, and media.

To encourage the uptake of the program within research groups at OPUS, all first year PhD students are partnered with a Research Buddy to inform their research from project inception. Early adoption of CCI at this stage reinforces the holistic integration of consumer voices throughout OPUS research projects.

Our research center is committed to strengthening the public voice and sharing power over decision making activities, acknowledging the inherent power imbalances that are often reported in consumer involvement programs. Typically, the hierarchal, rigid model of scientific research is favored in comparison to models of research led by the public, which further perpetuates this imbalance [7]. Nevertheless, progressing towards balance is possible when an organization demonstrates support towards a cultural shift that prioritizes community voices in their research practices [7] - a notion that our research center has committed to achieve.

At OPUS, we also acknowledge the importance of equality and inclusivity across our entire research center. The CCIP is underpinned by values that foster inclusivity and celebrate diversity. In the first instance, to ensure there is a balance of representative voices within the CCAG, we have stipulated that there be an equal number of researchers to non-researcher representatives within the group. Notably, at least three Consumer Advocates sit on the CCAG.

Furthermore, in our approach to address inequalities, the CCAG and consumer panel will liaise closely and often with OPUS’ Aboriginal and/or Torres Strait Islander Community Reference group, musculoskeletal specific, and general consumer organizations (e.g. MSK Australia, Health Issues Centre, and local support groups) to ensure a range of voices and perspectives are represented, consulted and involved regularly.

### Support structures

#### Formal training

Following recruitment, participants begin formal training in the form of an Orientation Workshop to prepare them on their consumer involvement journey and to network with fellow participants. Co-designed and co-led by consumer advocates, this workshop touches on the medical research landscape, the kinds of research projects conducted within OPUS, the research expertise within OPUS and the role of consumer and community involvement within OPUS and medical research.

Further training specific to each tier is ongoing, at their own pace, supported by the CCLO, and in some circumstances, ‘on the job’ with the researchers.

1. Does not require specific training as they receive regular information and as appropriate, surveys and resource material on consumer involvement. Members of this level are able to freely access training documents on the OPUS website which subsequently may encourage movement to different Tier levels.
2. Members are directed towards reference materials including guides on the document types to expect, what to look out for (spelling, grammar, clarity, visual appeal, comprehensibility, etc.) as well as some background information provided by the research group summarizing what the study is about and the document purpose. Additionally, health literacy training is provided to consumers to support and expand their health literacy knowledge. Notably their partnered researchers are also trained to ensure their work is more accessible by avoiding scientific jargon and learning to write in plain English.
3. While Research Buddies are expected to have similar Tier 2 document revision responsibilities, additional training and mentoring is centered around effectively contributing to meeting discussions. Research group leaders are expected to provide further background information specific to the study to consumers.
4. Prerequisite training is the same as that for Tier 3 in addition to guidance on committee management as part of their duties as a Consumer Advocate. These members have a good understanding of research and broader research themes to actively participate. OPUS management provides mentorship and support for this Tier.

#### Further support and opportunities

Consumer and researchers are provided regular opportunities to attend relevant workshops to further facilitate their involvement within the program. Consumers have ready access to the CCLO and the CCAG should they wish to discuss any grievances or would like additional support. OPUS hosts research networking events throughout the year in which consumer participants are invited to attend and contribute by co-presenting. This provides an opportunity for consumer participants to not only increase their knowledge base in musculoskeletal research, but to meet other OPUS researchers, embed more deeply in the team and to share experiences with other participants. Networking amongst peers is an important aspect that OPUS encourages through the online community board which has been built into the OPUS website. The community board and website serves multiple functions: allowing open communication between consumers, researchers, and OPUS management; a centralized ‘home base’ for consumers; and to encourage community and familiarity between users. Furthermore, there are opportunities (via routine biannual evaluations) for consumers to voice their needs to develop their research skills, which OPUS endeavors to support (e.g. learning about different research methods, developing skills to review research papers etc.).

#### Remuneration and compensation

It is important that the time and expertise of consumer and community members are valued and appropriately remunerated or otherwise acknowledged. Remuneration is based on time and type of contribution or tasks and follows a tiered approach (Table 3).

**Table 3.** Remuneration fee schedule in $AUD

Level	Task	Fees
Tier 1	• New updates	$0
	• Complete surveys	$0
Tier 2	• New updates	$0
	• Complete surveys	$0
	• Document review	$20/hr
Tier 3	• New updates	$0
	• Complete surveys	$0
	• Document review	$20/hr
	• Sitting fee	$25/hr
Tier 4	• New updates	$0
	• Complete surveys	$0
	• Document review	$20/hr
	• Sitting fee	$25/hr

The fee schedule has been created via consultation from other consumer involvement programs, Consumer’s Health Forum of Australia, researchers and in alignment with OPUS’ budget allowances. Consumers are remunerated according to the activities they chose to be involved in, including any relevant travel costs.

- **Tier 1:** In line with the expected time commitment at this level, Tier 1 consumers are volunteers and therefore, do not receive financial compensation. However, in recognition of their involvement, a certificate of participation is awarded at the end of their term of involvement.
- **Tier 2:** Consumers are compensated for the time taken to review documentation and provide feedback as we acknowledge that this is extra time invested in our research. Depending on the complexity of the documentation, reviewers receive gift cards of varying denominations appropriate to their contribution.
- **Tier 3:** Face-to-face and online meetings with researchers and students are remunerated on set hourly allocations and all travel expenses are reimbursed (this includes public transport tickets, parking and distance driven at 67c/km).
- **Tier 4:** Consumers participating in committee work are compensated for the time taken to prepare for and participate in meetings as well as for travel expenses (this includes public transport tickets, parking and distance driven at 67¢/km).

### Strategies to overcome barriers

Potential barriers and the strategies in place to overcome them are outlined in Table 4.

**Table 4:** Barriers and mitigation strategies

Barriers	Mitigation strategies
Lack of consumer sense of purpose	Comprehensive training about the CCIP, the role of consumers and responsibilities is provided. Importantly, a clear outline of the expectations of consumers, OPUS and their research partners are defined with all training material to be co-designed with Consumer Advocates to ensure appropriateness and relevancy. Consumers have ready access to the CCLO, the CCAG, the wider OPUS group and fellow consumer peers, to help support a lack of direction and purpose consumers may feel.
Lack of resources allocated	Continuous sourcing of funds are undertaken routinely by the CCAG and researchers to support consumer participation at OPUS. All participating researchers must build consumer involvement into their projects by incorporating consumer engagement as a budget line item in all grant submissions. Funding is managed by OPUS administration to ensure consistency throughout the program.
Poor communication between researchers and consumers	Ongoing training is provided to both researchers and consumers to ensure there are clear expectations of the roles within the CCIP. A direct line of communication is always available and should be maintained between the consumer and researcher; however, the Consumer and Community Liaison Officer is also available to facilitate any interactions and ensure an open line of communication between the researcher and consumer is maintained.
Poor understanding among researchers on effective consumer participation	Researchers that partake in the CCIP are required to attend training and workshops throughout their membership to ensure there is clear understanding and expectation of the role consumers have in their research.
Lack of consumer support and networks	This program has been built with multiple layers of communication to ensure consumers do not work in isolation. Points of contact include: the CCLO and support staff, an online network amongst fellow consumers and the CCAG, where issues can be escalated and monitored. Biannual evaluations will also be jointly developed and completed by consumers to assess their experience and involvement and to identify areas of improvement.
Lack of consumer interest/understanding of research project	A lay summary of the research project is provided to consumers to ascertain their interests. Once interest has been established, the researcher and consumer will meet (either face to face or via phone or zoom) to outline what the role entails and gives the opportunity for the consumer to seek further clarification about the project. The CCLO also monitors the frequency of interactions between researcher and consumer to ensure the consumer is satisfied with the support they are receiving and that the researcher is able to utilize the consumers’ skills and knowledge to their full capacity.

## Discussion

This paper demonstrates how the general recommendations provided by the NHMRC statement on consumer and community involvement can be appropriately actioned in an established research center like OPUS. There is great emphasis on building the capacity of consumers and ensuring their time and support is valued. This program leans heavily on building relationships with consumers and the community to: partake in the research development process; advocate for and educate communities on medical research; and advocate to policy makers to drive effective change. However, the responsibility and expectations of researchers must also be highlighted. Researchers must commit to the partnership by learning to communicate their latest developments in a comprehendible format, regularly engaging and consulting with their consumer partners and accepting and incorporating, where appropriate, the advice they receive from consumers into the research projects.

The appointment of a CCLO is paramount to this program by not only providing a centralized point of contact for consumers but informs the executive team at OPUS of consumer needs and support required. Additionally, this officer is key to building long-lasting meaningful relationships between consumers and researchers and by extension, the research center. In addition, the role of Consumer Advocates in mentoring and supporting newer members of the consumer panel ensures the sustainability of CCI involvement in OPUS’ research program.

By necessity, the implementation of this framework is an iterative and reflexive process, as it will evolve to accommodate consumer and researcher needs as they emerge. This will ensure adaptability to the needs of the consumer and community members and will enable research organizations like OPUS to better plan and design projects that will have greater impact for those that not only access health care services but the wider community (e.g. those who have not yet needed joint replacement may still benefit from the research carried out, and carers of people experiencing osteoarthritis). Engaging those who will benefit from OPUS research from the outset will ensure that the center asks the right questions, studies the outcomes that matter most to patients, and produce useful and relevant results that are more likely to be translated into policy and practice. Participating in a Consumer and Community Involvement Program provides consumers not only the opportunity to learn, grow and challenge their current knowledge base in health research but importantly, contributes to improving the health and wellbeing of people living with musculoskeletal conditions.

### Definitions

**Table:** 

Consumers	“Health Consumers are people who use health services, as well as their family and carers. This includes people who have used a health service in the past or who could potentially use the service in the future.” [8]
Community Member	“People who share a common interest or background (e.g. cultural, social, political, health, economic), or a particular public health or environmental exposure (e.g. an area of water contamination) but do not necessarily have a geographic association” [6].

## Data Availability

Not applicable.

## Abbreviations

NHMRC: National Health and Medical Research Council
CCI: Consumer and Community Involvement
OPUS: Optimization of outcomes, improved equity, cost effectiveness and patient selection
CCIP: Consumer and Community Involvement Program
CCAG: Consumer and Community Advisory Group
